# STAT3 and NF-κB are common targets for kaempferol-mediated attenuation of COX-2 expression in IL-6-induced macrophages and carrageenan-induced mouse paw edema

**DOI:** 10.1016/j.bbrep.2017.08.005

**Published:** 2017-08-26

**Authors:** Anandita Basu, Anindhya Sundar Das, Manoj Sharma, Manash Pratim Pathak, Pronobesh Chattopadhyay, Kaushik Biswas, Rupak Mukhopadhyay

**Affiliations:** a Cellular, Molecular and Environmental Biotechnology Laboratory, Department of Molecular Biology and Biotechnology, Tezpur University, Tezpur 784028, Assam, India; b Division of Pharmaceutical Technology, Defense Research Laboratory, Tezpur 784001, Assam, India; c Division of Molecular Medicine, Bose Institute, Kolkata 700054, India

**Keywords:** NF-κB, STAT3, Kaempferol, Polyphenol, Inflammation, Paw edema

## Abstract

Cycloxygenase-2 (COX-2) is the inducible isoform of cycloxygenase enzyme family that catalyzes synthesis of inflammatory mediators, prostanoids and prostaglandins, and therefore, can be targeted by anti-inflammatory drugs. Here, we showed a plant polyphenol, kaempferol, attenuated IL-6-induced COX-2 expression in human monocytic THP-1 cells suggesting its beneficial role in chronic inflammation. Kaempferol deactivated and prevented nuclear localization of two major transcription factors STAT3 and NF-κB, mutually responsible for COX-2 induction in response to IL-6. Moreover, STAT3 and NF-κB were simultaneously deactivated by kaempferol in acute inflammation, as shown by carrageenan-induced mouse paw edema model. The concomitant reduction in COX-2 expression in paw tissues suggested kaempferol’s role in mitigation of inflammation by targeting STAT3 and NF-κB.

## Highlights

• IL-6-induced COX-2 expression was attenuated by kaempferol in macrophages.
• The attenuation is attributed to simultaneous deactivation of STAT3 and NF-κB.
• The nuclear translocation of both transcription factors are prevented by kaempferol treatment.
• Kaempferol targets STAT3 and NF-κB and inhibits COX-2 expression to reduce carrageenan-induced mouse paw edema.

## Graphical abstract

**Figure:**
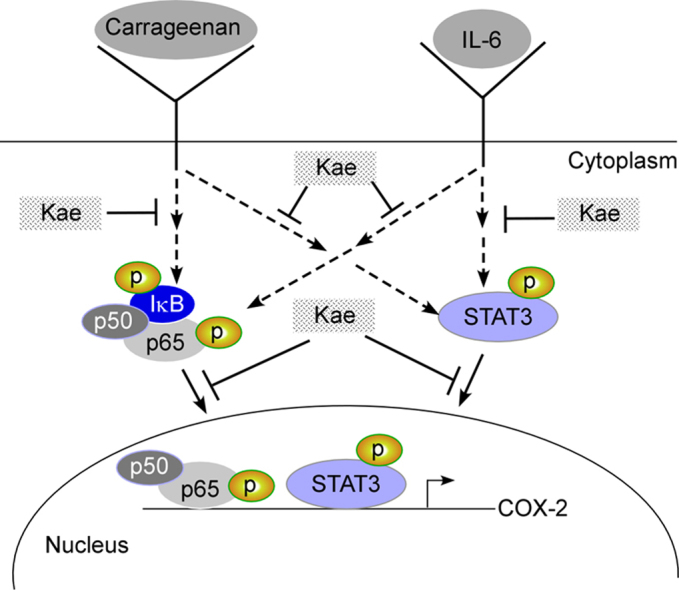

## 1 Introduction

Cycloxygenase-2 (COX-2) is one of the key enzymes that catalyzes the conversion of arachidonic acid to inflammatory mediators, prostaglandins and prostanoids [1]. Induction of COX-2 is common in a range of pathological conditions and in response to stimuli e.g. cytokines. Use of commercially available COX-2 inhibitors is common and shows beneficial effects against chronic inflammation-mediated diseases. Polyphenols, a ubiquitous group of plant metabolites and an integral part of human diet, can be a natural alternative to commercially available COX-2 inhibitors [2], [3]. Kaempferol (3,5,7-trihydroxy-2-(4-hydroxyphenyl)-4H-1-benzopyran-4-one) reported to possess a wide spectrum of potent pharmacological activities, including antioxidant, anti-inflammatory, antimicrobial, anticancer and anti-allergic actions [4]. It is available in dietary foods and beverages like tea, cabbage, beans, broccoli, tomato, grapes, strawberries and in medicinal plants like *Tilia spp* and propolis [4]. The inhibitory effect of kaempferol on various stimulus-induced COX-2 expressions has been studied. However, the effect of kaempferol on IL-6-induced COX-2 expression in macrophages, cells critical for initial response to inflammation, has not been reported.

Interleukin 6 (IL-6) is a pleiotropic cytokine that exhibits either a pro- or an anti-inflammatory effect in diverse cell types and conditions [5], [6]. IL-6 is associated with chronic inflammatory diseases such as coronary heart disease, Crohn’s disease, rheumatoid arthritis (RA), systemic-onset juvenile chronic arthritis (JCA), osteoporosis, psoriasis, multiple myeloma (MM), Castleman's disease, prostate carcinoma and systemic sclerosis [7], [8], [9], [10]. As a pro-inflammatory cytokine, it preferentially activates signal transducer and activator of transcription protein 3 (STAT3)-dependent gene expression [11]. Activation of STAT3 by IL-6 plays a crucial role in inflammation-induced disease pathogenesis [12], [13], [14]. IL-6 is known to activate COX-2 through its trans-signaling pathway [15], [16]. IL-6-STAT3-COX-2 axis was shown to be important in inflammation-induced malignancies and controlled by a positive feedback regulation [17], [18]. On the other hand, reports on IL-6-induced activation of NF-κB, another important transcription factor involved in inflammatory gene expression, is limited. Since NF-κB is known to be a regulator of COX-2 in response to other stimuli, it is important to understand the IL6-NF-κB-COX2 axis as well.

In addition, COX-2 expression plays a crucial role in acute inflammation. Carrageenan-induced mouse paw edema model is commonly studied to understand the efficacy of a compound against acute inflammation. Signaling processes that are targeted by these compounds demonstrate their mechanism of action for mitigation of inflammation. This study reports that in THP-1 cells, IL6-NF-κB-COX2 axis plays major role in addition to IL-6-STAT3-COX-2 axis and kaempferol-mediated inhibition of COX-2 is associated with simultaneous deactivation of both STAT-3 and NF-κB. Interestingly, kaempferol treatment led to deactivation of STAT3 and NF-κB and inhibition of COX-2 expression in carrageenan-induced mouse paw tissues suggesting its role in targeting these two transcription factors in inflammation independent of the type of stimulus.

## 2 Materials and methods

### 2.1 Cells and reagents

The human monocytic cell line THP-1 was purchased from the American Type Culture Collection (ATCC, USA) and was maintained in complete RPMI 1640 medium (Himedia, India) supplemented with 10% fetal bovine serum (Life Technologies, Gibco, USA) and penicillin/streptomycin (100 units/mL) (Life Technologies, Gibco, USA). Kaempferol (Sigma Aldrich, USA) was dissolved in dimethyl sulphoxide (DMSO) to a stock concentration of 100 mM. All antibodies used in this study were purchased from Cell Signaling Technology, USA. Recombinant IL-6, RIPA buffer and cDNA synthesis kit were obtained from Thermo Fisher Scientific, USA. Taq polymerase was from Biobharati Life Science, India. Phorbol-12-myristate-13-acetate (PMA), TriZol, S3I-201 and BAY11-7032 were obtained from Sigma Aldrich, USA.

### 2.2 Cell treatment

Around 0.5 × 10^6^–0.75 × 10^6^ THP-1 cells/well in 6-well plate or 8 × 10^6^cells/100 mm dish were differentiated to macrophages with 5 ng/mL PMA for 48 h. Cells were rested for 24 h in complete RPMI medium and treated with kaempferol for 6 h followed by induction with IL-6 for 2 h. All the treatments were done in 1% FBS-containing RPMI medium.

### 2.3 Semi-quantitative PCR analysis

The treated and control cells were harvested using TriZol (Life Technologies, USA) and RNA was isolated as per the manufacturer’s protocol. cDNA was obtained from the total RNA samples using Verso cDNA synthesis kit (Thermo Fisher Scientific, USA). Semi-quantitative PCR was performed with the cDNA samples using gene-specific primers. All primer sequences have been shown in Supplementary Table S1.

### 2.4 Western blot analysis

Cells were treated as stated above and harvested using RIPA buffer (Thermo Scientific, USA) containing protease and phosphatase inhibitor cocktails (Thermo Scientific, USA). Immunoblots were incubated overnight with specific primary antibodies at 4ᴼC followed by incubation with HRP-conjugated secondary antibody for 1 h. Developed blots were imaged and analyzed using Chemidoc XRS+ gel documentation system (Bio-Rad, USA).

### 2.5 Immunofluorescence studies

Cells were treated with kaempferol and IL-6 as stated earlier. After incubation, cells were washed with PBS and fixed with 4% paraformaldehyde for 15 min at room temperature. Fixed cells were permeabilized using 0.1% TritonX-100 (Sigma-Aldrich, USA) in PBS for 15 min followed by further washing. The cells were then incubated in blocking solution (PBS containing 0.1% Tween 20, 22.52 mg/mL glycine and 1% bovine serum albumin) for 1 h at room temperature. This was followed by incubation of the respective cell samples with phospho-STAT3 and NF-κB p65 antibody (at 1:100 and 1:400 dilution respectively), overnight at 4 °C. The cells were then washed and incubated with secondary antibody tagged with Alexa Fluor 488, for an hour at room temperature in dark. The cells were mounted with Prolong gold antifade mounting solution with DAPI (Thermo Fisher Scientific, USA) and observed with fluorescence microscope (Olympus model IX83, Japan).

### 2.6 Animal experiments

The animal experiment was carried out with approval from Institutional Animal Ethical Committee of Defense Research Laboratory, Tezpur. Thirty Swiss Albino mice were divided into five groups. Group I was maintained as negative control. Group II was intraperitonially (i.p.) injected with dimethyl sulphoxide (DMSO). The third group (Group III) was treated i.p. with indomethacin, a standard non-steroidal anti-inflammatory drug (10 mg/kg). The last two groups (Group IV and Group V) were injected with 1/10 (35 mg/kg) and 1/20 (17.5 mg/kg) of LD-50_mouse_ dose (350 mg/kg) of kaempferol, respectively using i.p. route. After 3 h of treatment in Group II-V, acute inflammation was promoted by the sub-cutaneous injection of 1% carrageenan solution in the sub-plantar space of the right hind paw, while left paws were injected with 50 µl normal saline. Time-dependent reduction in paw volume was measured at 0, 2 and 24 h as compared to control using a plethysmometer. Paw tissues were harvested for mRNA and protein expression using RT-PCR and western blot analysis, respectively. The primer sequences used for RT-PCR is given in Supplementary Table 1.

### 2.7 In silico study of upstream region of COX-2 gene

600 bp upstream sequence of COX-2 gene was used as feed to find predicted binding sites for NF-κB and STAT3 by TFBIND webtool (http://tfbind.hgc.jp/) [19].

### 2.8 Statistical analysis

Data were obtained with three independent experiments in most cases. One-way ANOVA and *post hoc* Bonferroni Comparison Test were used to determine the differences between groups of the data obtained from PCR and western blot by using GraphPad Prism (V6.0) for all the studies except the mouse paw volume data where Two-way ANOVA and Bonferroni *post hoc* test were used. P-values < 0.05 were considered statistically significant.

## 3 Results

### 3.1 Kaempferol inhibits IL-6-induced COX-2 expression in THP1 cells

**Fig. 1:**
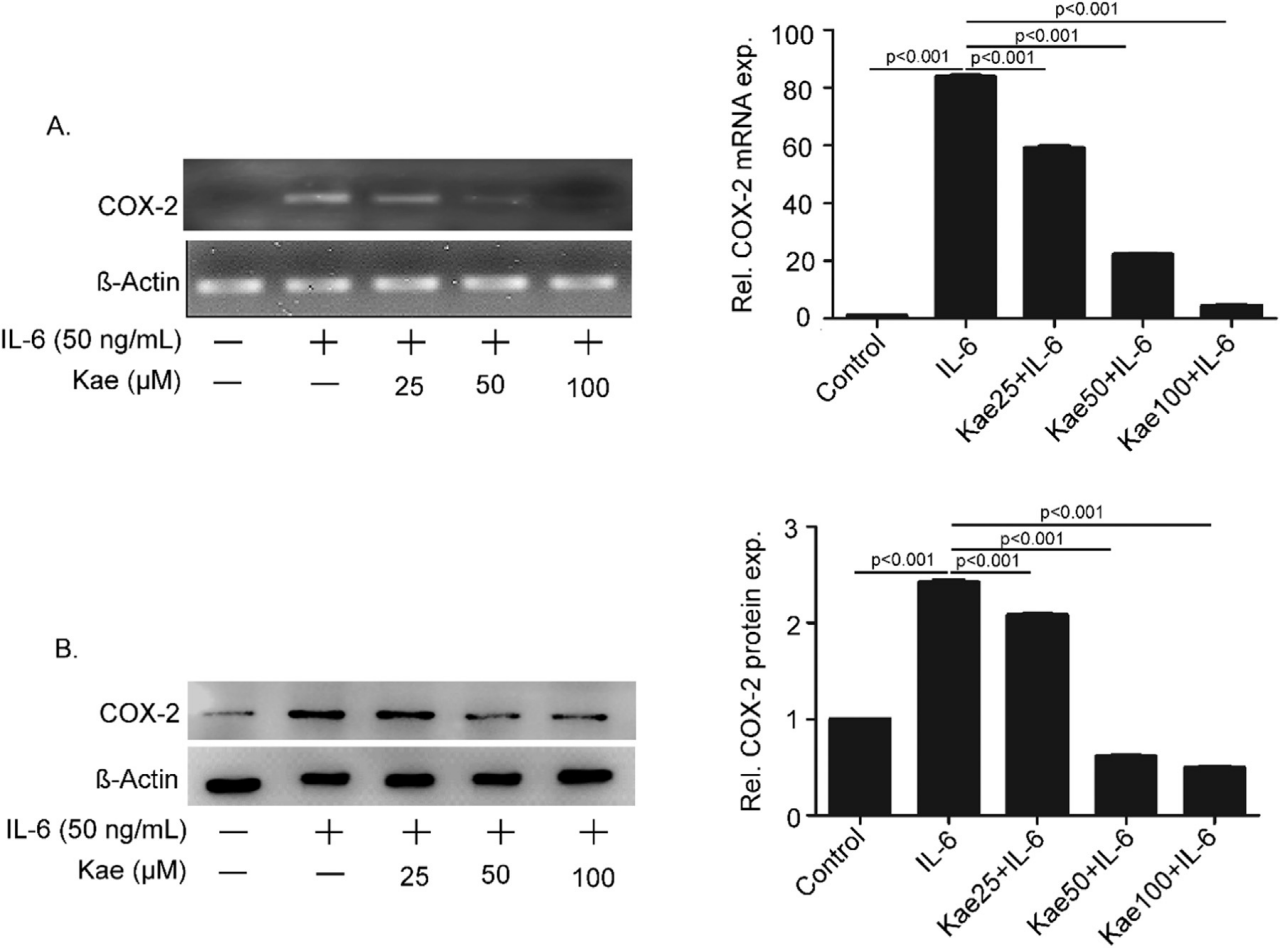
**Inhibition of IL-6-induced COX-2 expression in THP1 cells.** PMA-differentiated THP1 cells were pre-treated with kaempferol (Kae) at various concentrations followed by induction with IL-6 for 2 h. Expression of COX-2 mRNA and protein were studied using semi-quantitative PCR (**A**) and western blot using anti-COX-2 monoclonal antibody (Clone D5H5) (**B**). The band intensities were quantitated and represented as mean ± SEM of three independent experiments.

PMA-differentiated THP1 cells were pre-treated with kaempferol at three different concentrations, 25, 50 and 100 µM respectively for 6 h followed by IL-6 (50 ng/mL) induction for 2 h. Expression of COX-2 was measured in cells at mRNA and protein levels. RT-PCR using COX-2 gene specific primers suggested that IL-6-induced COX-2 mRNA expression was inhibited by kaempferol in a concentration- dependent manner (Fig. 1A). We further investigated the protein expression of COX-2 by western blot (Fig. 1B). Treatment with IL-6 led to 2.5 folds increase in COX-2 protein expression, which was inhibited by kaempferol in a dose-dependent fashion.

### 3.2 Activation of NF-κB, in addition to STAT3, is important for IL-6-induced COX-2 expression

**Fig. 2:**
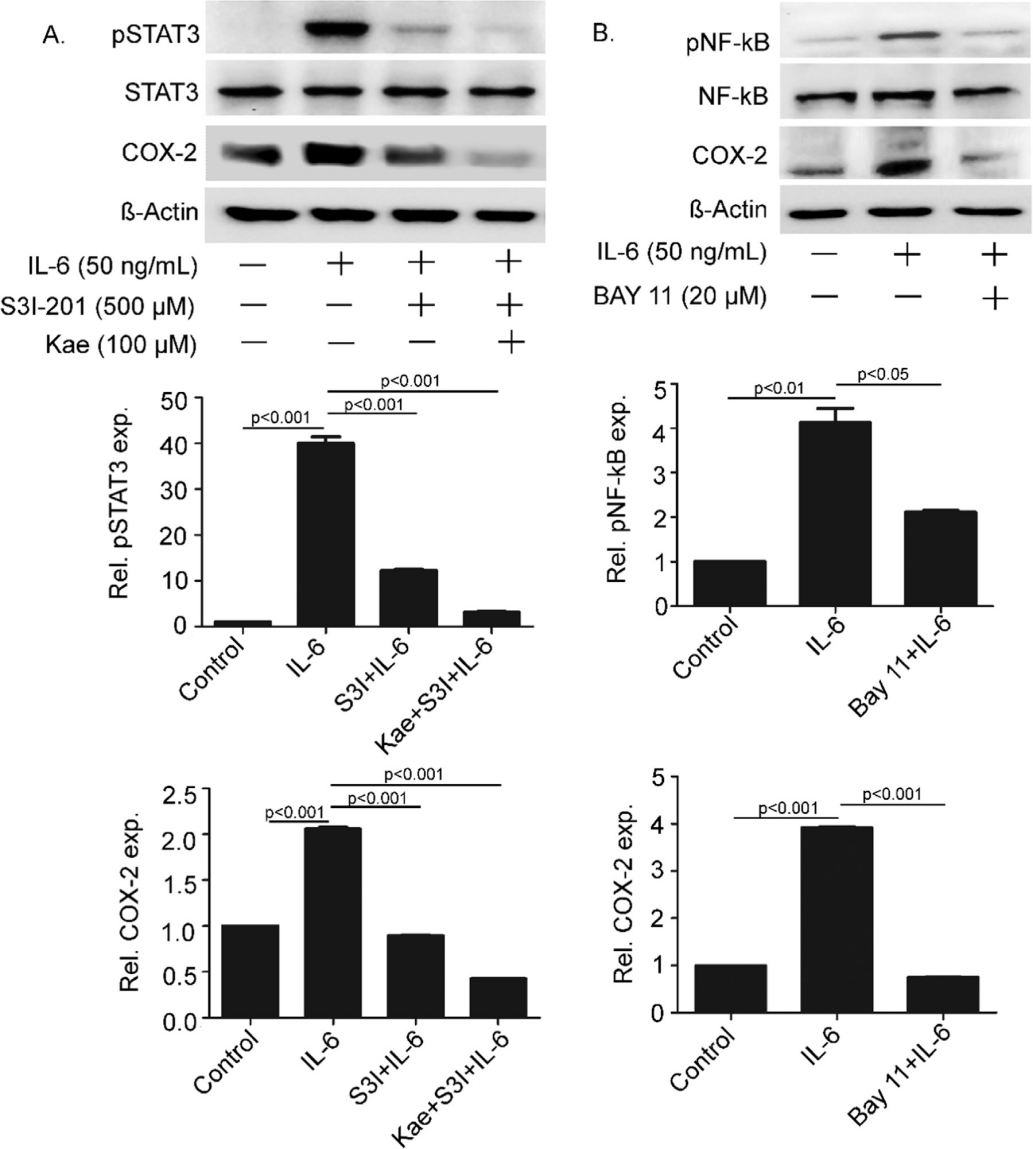
**STAT3 and NF-κB activations are required for IL-6-induced COX-2 expression.** THP-1 cells were pre-treated with S3I-201 alone or in combination with kaempferol (Kae) followed by induction with IL-6 (**A**). The cells were pre-treated with BAY-11 (**B**) before induction with IL-6. Expressions of p-STAT3 (Tyr 705), STAT3, p-NF-κB (Ser536), NF-κB and COX-2 were studied by western blots. The band intensities were quantitated and data are presented as mean ± SEM of three independent experiments.

Prior to understanding the mechanism of kaempferol-mediated attenuation of COX-2 expression it was important to find the pathway responsible for IL-6-induced COX-2 expression. IL-6 is reported for activation of STAT3 as a major transcription factor by the classical pathway in macrophages [20], [21]. STAT3-mediated induction of COX-2 expression in response to IL-6 has been reported in intestinal epithelial cells [20]. To study the role of STAT3 in IL-6-induced COX-2 expression in inflammatory cells, differentiated THP-1 cells were treated with IL-6 for 2 h. Western blot analysis with phospho-STAT3 (Tyr705) antibody showed activation of STAT3 without altering the total pool of STAT3 proteins (Fig. 2A; first and second panel). Expression of COX-2 protein was also induced significantly in IL-6-treated cells (Fig. 2A; third panel). STAT3 activation was attenuated when the cells were pre-treated with 500 μM of S3I-201, a specific inhibitor of STAT3, for 15 min. Concomitantly, COX-2 expression was reduced suggesting the role of STAT3 in IL-6-induced up regulation of COX-2. Surprisingly, treatment of cells with both S3I-201 and kaempferol led to further reduction in expression of COX-2 suggesting possible role of a different pathway in addition to JAK-STAT pathway (Fig. 2A). Till date, there is no report on the role of IL-6-mediated activation of NF-κB for COX-2 expression in macrophages. To study whether NF-κB was also involved in IL-6-mediated COX-2 expression, cells were treated with IL-6 for 2 h. IL-6-induced activation of NF-κB as shown by four folds increase in the expression of phosphorylated form (Ser 536) of NF-κB p65 isoform (Fig. 2B; first panel). The expression of non-phosphorylated form of NF-κB remained unaltered (Fig. 2B; second panel). The COX-2 protein expression was induced to four folds as a result of this induction (Fig. 2B; third panel). Interestingly, pre-treatment of the cells with 20 μM BAY-11, a specific inhibitor of NF-κB activation, reduced COX-2 expression significantly, suggesting a regulatory role of NF-κB activation for the expression of COX-2 in IL-6-induced inflammatory macrophages (Fig. 2A; first and third panel). To study available NF-kB and STAT3 binding sites in the promoter region of COX-2, 600 bp upstream sequence of the gene was analyzed using TFBIND webtool. Multiple predicted sites for both the transcription factors were present in the region (Supplementary Table S2). Together, these experiments suggested critical roles of both NF-κB and STAT3 for IL-6-induced activation of COX-2.

### 3.3 Kaempferol inhibited IL-6-induced activation and nuclear translocation of NF-κB and STAT3

**Fig. 3:**
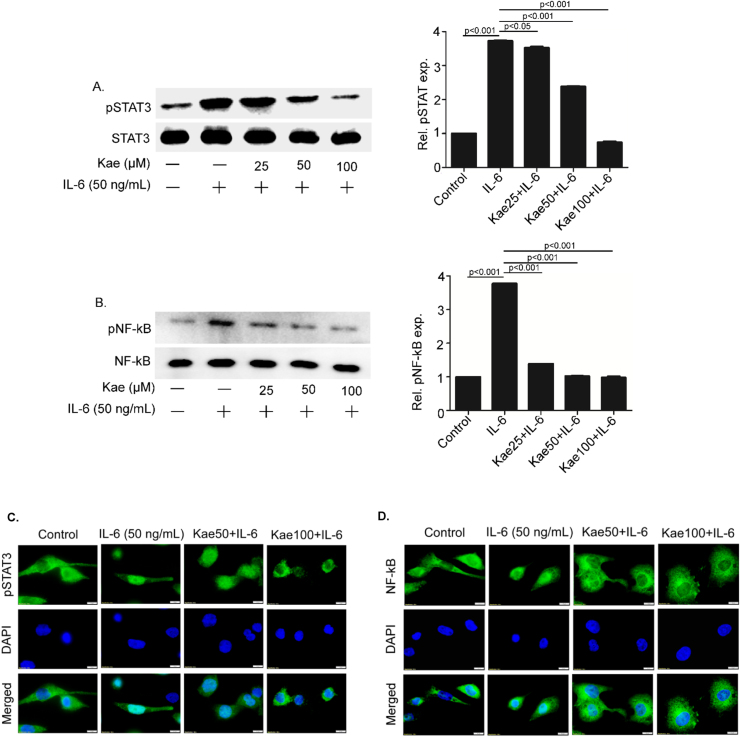
**Kaempferol inhibits the activation and nuclear translocation of STAT3 and NF-κB.** THP1 cells pre-treated with kaempferol (Kae) at various concentrations were induced with IL-6 for 2 h. Phosphorylation status of STAT3 and NF-κB was studied using specific antibodies **(A** and **B).** Data are presented as mean ± SEM of three independent experiments. Immunofluorescence images to study the effect of two different concentrations of kaempferol (50 and 100 μM) treatment on nuclear translocation of STAT3 (**C**) and NF-κB **(D).**

As IL-6-mediated activation of both NF-κB and STAT3 was important for COX-2 induction, we next studied the inhibitory effect of kaempferol on these two transcription factors. Western blot analysis showed that pre-treatment with kaempferol inhibited IL-6-induced phosphorylation of both STAT3 (at Tyr705) and NF-κB p65 (at Ser 536) (Fig. 3A and B). While inhibition of NF-κB p65 phosphorylation was highly significant at a very low concentration of kaempferol (25 µM), phosphorylation of STAT3 was inhibited only at higher concentration of kaempferol at 50 and 100 µM.

To understand the inhibitory mechanism of kaempferol on NF-κB and STAT3 activation, nuclear translocation of these two transcription factors were studied. PMA-differentiated THP1 cells were pre-treated with kaempferol at concentrations of 50 and 100 μM respectively for 6 h followed by IL-6 induction for 2 h. Immunostaining of the cells using anti-phospho STAT3 and anti-NF-κB p65 antibodies confirmed increased translocation of both STAT3 and NF-κB p65 in the nucleus of the IL-6-treated cells (Fig. 3C and D). However, nuclear translocation was significantly inhibited in kaempferol-treated cells in a concentration gradient manner (Fig. 3C and D). This study implied that kaempferol-mediated inhibition of nuclear translocation of both STAT3 and NF-κB is responsible for attenuation of IL-6-induced COX-2 protein expression in THP1 cells.

### 3.4 Kaempferol-mediated inhibition of COX-2 expression in mouse paw edema model

**Fig. 4:**
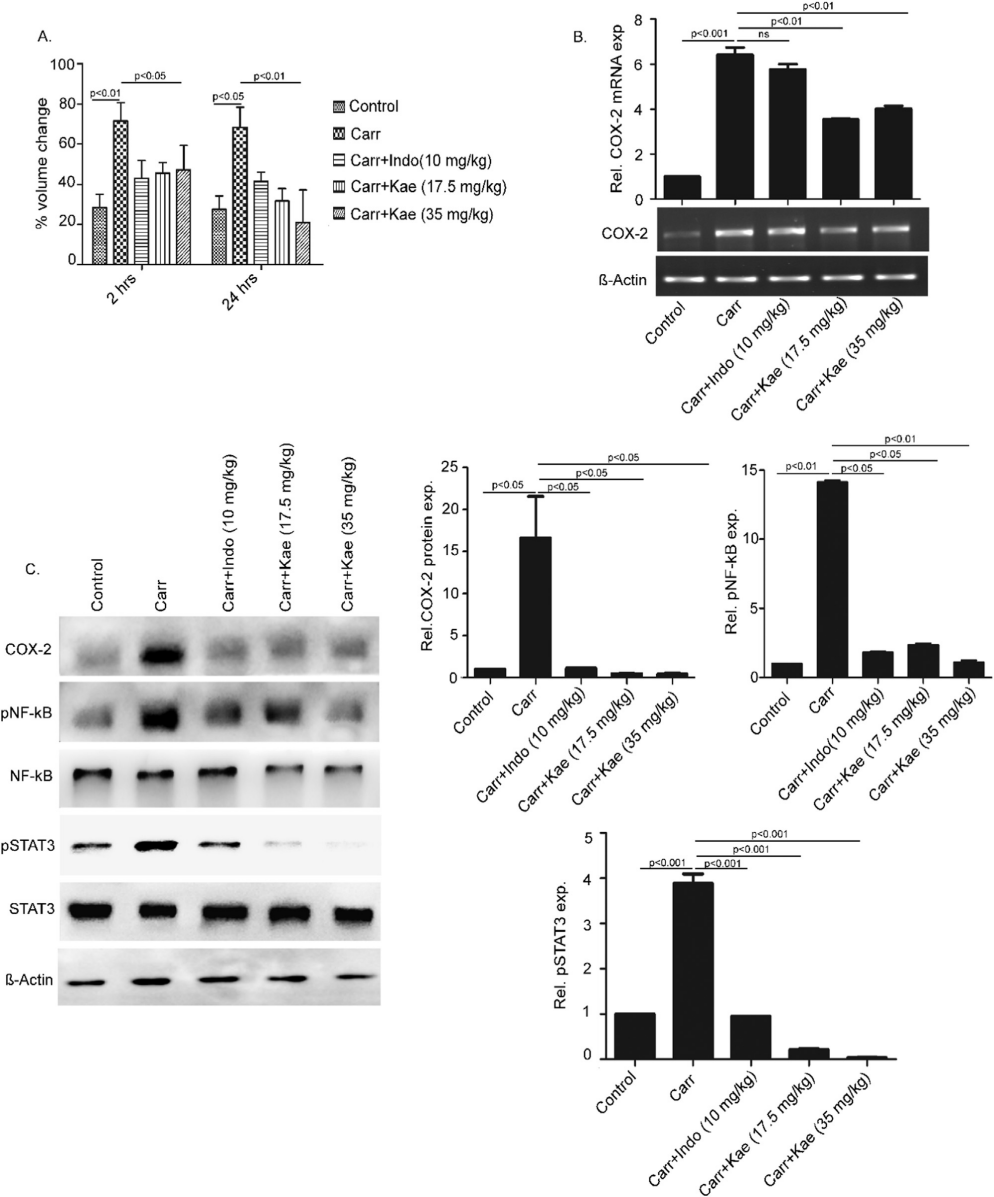
**Kaempferol reduces carrageenan-induced mouse paw edema volume alongside inhibition of NF-κB, STAT3 and COX-2 activation.** The mean percent change in paw volume of different groups of mice was measured using a plethysmometer at 2 and 24 h (**A**). Harvested paw tissues were analyzed for mRNA expression of COX-2 using semi-quantitative RT-PCR (**B**). The paw lysates were analyzed for expression of COX-2 and phospho and non-phosphorylated forms of STAT3 and NF-κB (**C**). Data are presented as mean ± SEM (n = 6).

Our data suggested that NF-κB and STAT3 are crucial targets of kaempferol for the inhibition of IL-6-induced COX-2 expression in THP-1 cells. A recent report suggested the inhibition of LPS-induced COX-2 expression by kaempferol by targeting NF-κB in macrophage cell lines [22]. To understand whether NF-κB and STAT3 are common targets of kaempferol-mediated inhibition of COX-2 expression, we decided to study carrageenan-induced mouse paw edema model. Among five groups of mice, we treated one group with indomethacin, a non-steroidal anti-inflammatory compound and two groups with different doses of kaempferol. The carrageenan-induced increase in paw volume was significantly reduced by kaempferol after 2 and 24 h (Fig. 4A). The reduction due to kaempferol was comparable with indomethacin at 2 h and even greater after 24 h suggesting its role as a general anti-inflammatory compound. COX-2 mRNA expression in the treated paw tissue samples was studied using RT-PCR and it was found to be down-regulated in samples treated with kaempferol (Fig. 4B). The western blot analysis confirmed the inhibitory effect of kaempferol on COX-2 protein expression which was drastically reduced in paw tissues of animals treated with kaempferol (Fig. 4C; first panel). Carrageenan-induced phosphorylation of both NF-κB (at Ser 536) and STAT3 (at Tyr 705) was inhibited by kaempferol as suggested by western blots with phophorylated antibodies against these transcription factors. The total levels of these two transcription factors remained largely unaltered. Together these data emphasized the coordinated role of NF-κB and STAT3 to induce COX-2 expression in IL-6-activated macrophages or carrageenan-induced mouse paw tissues. Strikingly, kaempferol targets both these transcription factors for the transcriptional regulation of COX-2 in these conditions.

## 4 Discussion

Dietary polyphenols are the most important bioactive molecules from plants that offer wide range of beneficial effects in human [23]. By virtue of their anti-inflammatory properties, polyphenols show significant effects against inflammation-induced disease pathogenesis [23], [24]. COX-2 is directly responsible for induction of prostaglandin production leading to inflammation. So, targeting COX-2 by polyphenols can be a logical therapeutic strategy to limit inflammation. Polyphenols e.g. resveratrol, saponin, luteolin, sulforaphane, oroxylin A were reported to attenuate COX-2 expression induced by LPS, IL-1β or TNF-α [25], [26], [27], [28]. However, the mechanism of IL-6-induced COX-2 expression in macrophages and its attenuation by kaempferol, a well-known anti-inflammatory and anti-oxidant polyphenol, is unknown in macrophages. Macrophage IL-6 signaling has significant implications in disease pathogenesis [29]. IL-6 induces expression of acute phase proteins which are produced as initial response towards external inflammatory stimuli, though its role in development of chronic inflammation is better characterized [30]. IL-6 produced by endothelial cells is the critical mediator for transition of acute to chronic phase of inflammation. Combination of IL-6 and its soluble receptor (sIL-6R) interacts with gp130 that leads to neutrophil apoptosis by macrophages. On the other hand, this interaction induces MCP-1 to recruit additional macrophages to the site of inflammation [31]. Elevated levels of IL-6 were found in chronic inflammatory diseases such as arthritis and colitis suggesting importance of the cytokine as a marker for chronic inflammatory diseases. Our study showed that IL-6-mediated over expression of COX-2 required activation of STAT3 and NF-κB, as inhibition of both transcription factors by specific inhibitors led to down-regulation of COX-2. Activation of STAT3 is a part of the classical signaling pathway of IL-6, but activation of NF-κB by IL-6 in inflammatory cells is not reported. We found that COX-2 expression was attenuated further in cells pre-treated with STAT3 inhibitor, S3I-201and kaempferol, than cells pre-treated with only S3I-201. We reasoned that attenuation of residual COX-2 expression might be attributed to inhibition of NF-κB activation by kaempferol. We demonstrated that inhibition of NF-κB activation by a specific inhibitor, BAY-11, reduced COX-2 expression significantly (Fig. 2B). Interestingly, IL-6 is one of the cytokines along with others e.g. TNF-α, IL-1 and IL-8, that are regulated by NF-κB and plays critical role in immune response [32]. Kaempferol targeted IL-6-induced phosphorylation of STAT3 at Tyr 705 and p65 subunit of NF-κB at Ser 536 for the attenuation of COX-2 expression. The expression of total STAT3 and NF-κB remained unaltered; however, their translocation to the nucleus was inhibited leading to transcriptional down-regulation of COX-2 expression. IL-6 phosphorylates the Ser 536 residue located at the transactivation domain (TAD) of p65 subunit of NF-κB. This phosphorylation event is well characterized for its role in enhanced transcriptional activity of NF-κB. Inhibition of Ser 536 phosphorylation by kaempferol for attenuation of COX-2 expression suggested the significance of this phosphorylation in transcriptional up-regulation of COX-2 in response to IL-6. Kaempferol-mediated inhibition of phosphorylation of two distinct sites, Tyr 705 of STAT3 and Ser 536 of p65 subunit of NF-kB might indicate inhibition of a common upstream event responsible for activation of these two transcription factors. It is tempting to speculate that kaempferol targets JAK which has been shown to activate IKK, the kinase for NF-κB and STAT3 under different conditions [20], [33]. However, further study needs to be performed to validate this point.

The collaboration between STAT3 and NF-κB for regulation of inflammatory gene expression has been proposed earlier [34]. This could be achieved by binding of these transcription factors to a promoter individually or following mutual interaction. Physical interactions between NF-κB family members, especially p65 with STAT3 have been reported in human mesangial cells and hepatocellular carcinoma cell lines [35], [36]. Binding sites of STATs are reported to be in close proximity with the binding sites of some other transcription factors, like the nuclear factor κB (NF-κB), which cooperate with the STATs in their function of co-regulating gene expression [20]. Further probe on the upstream events of the phosphorylation of these transcription factors might shed light on their interaction, however, it may be speculated that the coordination between STAT3 and NF-κB is critical for the upregulation of COX-2 in response to IL-6.

Kaempferol regulated the activation of both STAT3 and NF-κB for attenuation of COX-2 expression in response to IL-6. Kaempferol has been reported to inhibit NF-κB for attenuation of LPS-induced COX-2 expression [22]. However, to understand if STAT3 and NF-κB can be targeted simultaneously by kaempferol and attenuate COX-2 expression in other conditions, carrageenan-induced mouse paw edema model was studied. The swelling of hind paw of mice injected with carrageenan has been associated with production of prostaglandins and elevated levels of COX-2 [37]. We found a single dose of kaempferol treatment reduced the hind paw volumes significantly and COX-2 expression at both mRNA and protein levels. Interestingly, in the paw tissues, carrageenan-induced activation of STAT3 and NF-κB was inhibited by kaempferol suggesting the ability of the polyphenol to target both these transcription factors irrespective of their activator stimuli.

In conclusion, kaempferol-mediated attenuation of COX-2 expression in IL-6-stimulated inflammatory cells or carrageenan-induced mouse paw tissues requires deactivation of both STAT3 and NF-κB. The nuclear localization of these transcription factors is prevented suggesting the polyphenol’s critical role in regulation of the downstream genes important for both acute and chronic inflammation.

## Footnotes

Appendix B Supplementary data associated with this article can be found in the online version at doi:10.1016/j.bbrep.2017.08.005.
