# Supplementary material for: STAT3 and NF-κB are common targets for kaempferol-mediated attenuation of COX-2 expression in IL-6-induced macrophages and carrageenan-induced mouse paw edema
Source: Biochem Biophys Rep. 2017 Aug 26;12:54–61. doi: 10.1016/j.bbrep.2017.08.005 (PMC5613220; doi:10.1016/j.bbrep.2017.08.005)
Supplement: Supplementary file 2 — Supplementary material [file mmc2.docx]

**Supplementary Table S1:**

| **Gene** | **Sequence (5΄-3΄)** | **Amplicon size** |
| --- | --- | --- |
| **COX-2 (human_forward)** | GTTCCAGACAAGCAGGCTAATA | **78** |
| **COX-2 (human_reverse)** | CCACTCAAGTGTTGCACATAATC |  |
| **COX-2 (mouse_forward)** | TGCTGTACAAGCAGTGGCAA | **141** |
| **COX-2 (mouse_ reverse)** | GCAGCCATTTCCTTCTCTCC |  |
| **Beta actin (human_forward)** | AGAGCTACGAGCTGCCTGAC | **184** |
| **Beta actin (human_reverse)** | AGCACTGTGTTGGCGTACAG |  |
| **Beta actin (mouse_forward)** | AGAGGGAAATCGTGCGTGAC | **138** |
| **Beta actin (mouse_reverse)** | CAATAGTGATGACCTGGCCGT |  |
