# Supplementary material for: STAT3 and NF-κB are common targets for kaempferol-mediated attenuation of COX-2 expression in IL-6-induced macrophages and carrageenan-induced mouse paw edema
Source: Biochem Biophys Rep. 2017 Aug 26;12:54–61. doi: 10.1016/j.bbrep.2017.08.005 (PMC5613220; doi:10.1016/j.bbrep.2017.08.005)
Supplement: Supplementary file 3 — Supplementary material [file mmc3.docx]

**Supplementary Table S2: Predicted binding sites for NF-kB and STAT3 on the 600 bp upstream sequence of COX-2 transcription start site using TFBIND webtool (**[**http://tfbind.hgc.jp/**](http://tfbind.hgc.jp/)**)**

| **Matrix** | **TF label** | **Similarity score** | **Position on input strand** | **Strandness** | **Consensus Sequence*** | **Subsequence from the input sequence at the position - corresponding to the consensus sequence** |
| --- | --- | --- | --- | --- | --- | --- |
| **NFkB** | | | | | | |
| M00052 | V$NFKAPPAB65_01 | 0.817006 | 118 | (+) | GGGRATTTCC | AGGGGATTCC |
| M00052 | V$NFKAPPAB65_01 | 0.952096 | 119 | (+) | GGGRATTTCC | GGGGATTCCC |
| M00052 | V$NFKAPPAB65_01 | 0.851976 | 119 | (-) | GGGRATTTCC | GGGGATTCCC |
| M00052 | V$NFKAPPAB65_01 | 0.807425 | 120 | (-) | GGGRATTTCC | GGGATTCCCT |
| M00052 | V$NFKAPPAB65_01 | 0.775329 | 343 | (+) | GGGRATTTCC | GGGGACTACC |
| M00052 | V$NFKAPPAB65_01 | 0.824192 | 344 | (+) | GGGRATTTCC | GGGACTACCC |
| M00052 | V$NFKAPPAB65_01 | 0.794012 | 345 | (-) | GGGRATTTCC | GGACTACCCC |
| M00052 | V$NFKAPPAB65_01 | 0.829701 | 381 | (+) | GGGRATTTCC | CTGGGTTTCC |
| M00052 | V$NFKAPPAB65_01 | 0.783234 | 382 | (+) | GGGRATTTCC | TGGGTTTCCG |
| **STAT3** | | | | | | |
| M00225 | V$STAT3_01 | 0.713396 | 269 | (+) | NGNNATTTCCSGGAARTGNNN | AGACATCTGGCGGAAACCTGT |
| M00225 | V$STAT3_01 | 0.712238 | 269 | (-) | NGNNATTTCCSGGAARTGNNN | AGACATCTGGCGGAAACCTGT |

**S = C or G, R = A or G, N = any base pair.*
